# Conservative Management of Ulnar Collateral Ligament Injury in a Throwing Elbow Complicated by Ulnar and Median Neuropathy: A Case Report

**DOI:** 10.7759/cureus.93061

**Published:** 2025-09-23

**Authors:** Hiroki Nakagawa, Masashi Kawabata, Hidenori Futamura, Ryo Futamura, Katsumasa Sugimoto

**Affiliations:** 1 Department of Rehabilitation, Nagoya Sports Clinic, Nagoya, JPN; 2 Department of Rehabilitation, Kitasato University School of Allied Health Sciences, Sagamihara, JPN; 3 Department of Orthopaedics, Nagoya Sports Clinic, Nagoya, JPN

**Keywords:** baseball pitcher, hydrodissection (hydrorelease), median nerve, nerve mobilization, return to play, ulnar collateral ligament, ulnar nerve, ultrasonography

## Abstract

Ulnar collateral ligament (UCL) injury is a common cause of medial elbow pain in baseball pitchers. Concomitant ulnar nerve involvement is common, whereas median nerve dysfunction may further impair dynamic stability via reduced flexor digitorum superficialis (FDS). However, reports on the conservative management of both nerves with neuromuscular re-education remain limited. A 17-year-old high school baseball pitcher presented with medial elbow pain, weakness in ulnar nerve-innervated muscles, and sensory disturbance after he pitched. Magnetic resonance imaging (MRI) and ultrasonography revealed UCL injury with valgus laxity. Examination showed tenderness over the cubital tunnel (ulnar nerve) and along the median-nerve course with neurological deficits (sensory loss, weakness) of the right elbow. Initial physiotherapy consisted of ultrasound-guided mobilization of the cubital tunnel and median nerve branches, combined with nerve-gliding techniques and real-time ultrasonography feedback for targeted activation of the FDS and flexor carpi ulnaris. Although early treatment improved pain and reduced spasms, symptoms recurred during throwing progression. Subsequent ultrasound-guided hydrodissection (hydrorelease) of the cubital tunnel and median nerve branch near the FDS, followed by continued neuromuscular re-education, resulted in complete pain relief, restoration of muscle strength, and improved elbow stability. Ultrasonography confirmed reduced valgus widening during FDS contraction. The athlete successfully returned to competitive pitching two months post-injury and remained symptom-free at four months. This patient highlights that ultrasound-guided physiotherapy, including manual release, hydrodissection, and functional re-education targeting both the ulnar and median nerves, can restore dynamic stabilization of the elbow and facilitate an early return to play in patients with UCL injuries with concomitant neuropathy.

## Introduction

Elbow injuries related to overhead throwing are among the most common sports disorders in baseball players, with a reported prevalence of 17%-69% [1]. During pitching, valgus torque can reach approximately 64 Nm [2], which exceeds the ultimate tensile strength of the ulnar collateral ligament (UCL) [3]; accordingly, UCL injury is widely regarded as the principal cause of medial elbow pain in overhead athletes [3]. Medial elbow pain often coexists with ulnar neuropathic symptoms, mainly attributed to traction or compression of the ulnar nerve during the late cocking phase [4]. In addition, the median nerve provides articular branches around the medial elbow ligaments and may also contribute to medial elbow pain [5].

Dynamic stabilization of the UCL is provided by the flexor-pronator muscles, particularly the ulnar nerve-innervated flexor carpi ulnaris (FCU) [6] and the median nerve-innervated flexor digitorum superficialis (FDS) and pronator-flexor group [7]. Therefore, comprehensive interventions that target these neuromuscular structures are critical for protecting the ligament, facilitating return to play, and preventing recurrence. However, conservative strategies for patients who present with concurrent ulnar and median nerve symptoms together with UCL injury remain insufficiently established.

Here, we present the case of a high-school baseball pitcher with a UCL injury accompanied by both ulnar and median nerve symptoms who achieved early return to play through a combined program of ultrasound-guided manual therapy and neural mobilization.

## Case presentation

**Figure 1:**
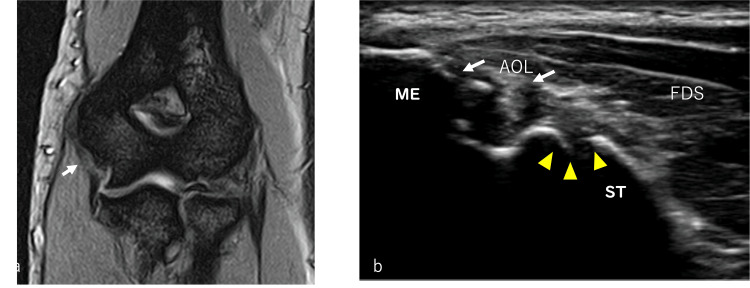
Magnetic Resonance Imaging (MRI) and ultrasound images of the injured site (a) MRI; (b) Ultrasound. On the affected side, an avulsion of the medial epicondyle and injury to the ulnar collateral ligament (UCL) can be observed (arrows). Yellow arrowhead: joint space. ME: medial epicondyle; ST: medial epicondyle to the sublime tubercle; FDS: flexor digitorum superficialis; AOL: anterior oblique ligament

A 17-year-old male high-school baseball pitcher presented with a three-week history of discomfort in the medial aspect of the right elbow that began during pitching practice, followed after training by weakness in ulnar nerve-innervated hand muscles (e.g., interossei, adductor pollicis). The next day, while pitching in a game, he developed medial elbow pain during the transition from late cocking to ball release. He was evaluated at our hospital the following day and diagnosed with a right UCL injury with involvement of both the ulnar and median nerves (Figure 1). He underwent three weeks of low-intensity pulsed ultrasound therapy and relative rest, after which physical therapy was initiated.

Physical assessment

**Figure 2:**
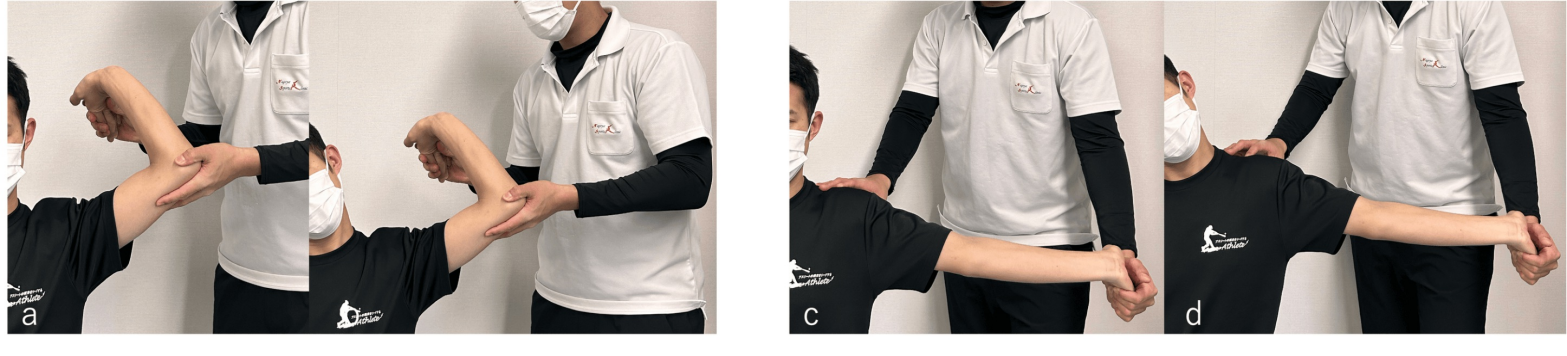
Nerve tension tests (a, b) Ulnar nerve tension test (from start to end); (c, d) Median nerve tension test (from start to end)

At the initial evaluation, tenderness was noted at the medial epicondyle, the sublime tubercle, and within the cubital tunnel. He reported tightness from the distal one-third of the upper arm to the proximal one-third of the forearm at terminal elbow flexion and extension. Orthopedic testing revealed positive valgus stress at 90° and 30° of elbow flexion. Upper-limb neurodynamic tests for the ulnar and median nerves provoked pain (Figure 2) [8]. Sensory testing demonstrated hypoesthesia in both ulnar and median nerve distributions. Grip strength and manual muscle testing indicated weakness in ulnar- and median-nerve-innervated muscles (Table 1).

**Table 1:** Physical examination findings of the patient ROM, range of motion; NRS, Numerical Rating Scale; NTT, Nerve Tension Test; MMT, Manual Muscle Testing; AOL, anterior oblique bundle of the ulnar collateral ligament; PT, pronator teres; FDS, flexor digitorum superficialis; FCU, flexor carpi ulnaris; Cub-tun, cubital tunnel syndrome; ST-arc, struther’s archade; ODM, opponens digiti minimi; ADM, abductor digiti minimi; FDP, flexor digitorum profundus; UnAF, unaffected side; AF, affected side; Int, Intervention; RTS, return to sports; US-FB, ultrasound feedback

Parameter		1st session		2nd session	3rd session	4th session
Time point		(Pre Int)	(Post Int)	(week2)	(week5)	(week8)
Elbow joint ROM	Extension	−3°	0°	→	→	→
	Flexion	135°	140°	→	→	→
Tenderness	AOL	+	−	−	−	−
	PT	+	−	−	−	−
	FDS	+	−	+	−	−
	FCU	+	−	+	−	−
Pain during valgus stress (NRS: /10)		9	0	5	0	0
Pain during NTT (NRS: /10)	Ulnar	9	0	5	1	0
	Median	8	0	5	1	0
Sensory (normal: 10)	Ulnar	6	10	8	10	10
	Median	7	10	8	10	10
Tinel sign (NRS: /10)	Cub-tun	8	0	3	0	0
	ST-arc	5	0	3	0	0
MMT (grade 0–5)	FCU	3	4	3	4	5
	ODM	3	4	5	5	5
	ADM	3	4	5	5	5
	FDS	3	4	5	5	5
	PT	4	4	5	5	5
	FDP	4	4	3	4	5
Grip strength	UnAF	45kg				
	AF	35kg	40kg	35kg	45kg	50kg
Playing level		No throw	30m	30m	Partial RTS	Full RTS
Intervention		Perineural mobilization → → →				
		Long-and Short-axis nerve gliding		Hydrodissection (Hydrorelease)	Long-and Short-axis nerve gliding	
		Muscle contraction with US-FB → → →				

Ultrasonographic evaluation

Under gravity-induced valgus stress, the medial joint space of the affected elbow was widened compared with the unaffected side (Video 1). Moreover, during stress, voluntary FDS contraction did not reduce medial joint gapping (Video 2). Sonopalpation-compressing the ulnar and median nerves and surrounding tissues with the edge of the ultrasound probe-reproduced radiating pain identical to the symptoms experienced during throwing.

**Video 1:** Valgus stress test under body weight Widening of the joint space on the affected side.

**Video 2:** Valgus stress test under body weight with flexor digitorum superficialis ontraction No change in joint space distance on the affected side.

Initial intervention

**Figure 3:**
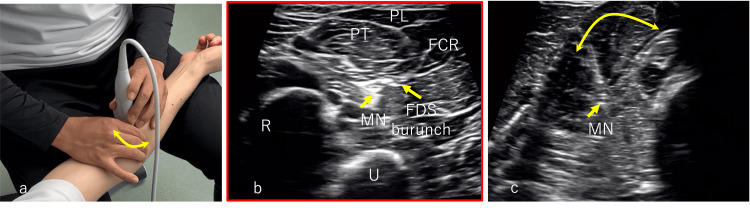
Ultrasound-guided exercise of the right median nerve (a) Approach image,(b) Ultrasound image at rest,(c) Ultrasound image during manual intervention to enhance neural gliding.Yellow double arrow: Direction of manual manipulation (displacement of the FDS and surrounding area of the median nerve in the short-axis direction relative to the forearm).Yellow single arrow: deformation of the median nerve FDS branch and surrounding muscles induced by manual manipulation.R, radius; U, ulna; PT, pronator teres; PL, palmaris longus; FCR, flexor carpi radialis; FDS, flexor digitorum superficialis; FCU, flexor carpi ulnaris; FDP, flexor digitorum profundus; MN, median nerve; FDS branch, branch of the flexor digitorum superficialis

For ulnar-nerve symptoms, ultrasound-guided mobilization of the FCU and the adjacent ulnar nerve at the cubital tunnel was performed for approximately five minutes, followed by 10 repetitions of longitudinal and transverse nerve slider/tensioner maneuvers to improve gliding between the FCU and the ulnar nerve (Video 3). Ultrasound biofeedback training was then conducted, during which the patient repeatedly contracted the FCU, opponens digiti minimi, and abductor digiti minimi with visual confirmation of activation. Similar mobilization and longitudinal/transverse slider/tensioner maneuvers were applied to the median nerve (Figure 3, Video 4), followed by ultrasound biofeedback training of the FDS, pronator teres, and flexor digitorum profundus. Immediately after the initial session, muscle spasm and tenderness improved (Table 1).

**Video 3:** Ultrasound-guided exercise of the right ulnar nerve Approach image. Red double arrow: direction of manual manipulation (short-axis movement of the tissue surrounding the ulnar nerve relative to the humerus). Ultrasound image. Red double arrow: direction of manual manipulation (short-axis movement around the ulnar nerve relative to the humerus). Red single arrow: sliding improvement between the ligamental capsule complex and ulnar nerve or flexor carpi ulnaris (FCU). O: olecranon; ST: medial epicondyle to the sublime tubercle

**Video 4:** Ultrasound-guided exercise of the right median nerve approach image and ultrasound image. R, radius; U, ulna; PT, pronator teres; PL, palmaris longus; FCR, flexor carpi radialis; FDS, flexor digitorum superficialis; FCU, flexor carpi ulnaris; FDP, flexor digitorum profundus; MN, median nerve; FDS branch, branch of the flexor digitorum superficialis

Recurrence and additional intervention

At the start of the second physical therapy session, the patient had resumed light throwing (up to ~30 m); however, medial elbow pain recurred. Re-examination revealed renewed stretch-induced pain of the ulnar and median nerves along with weakness in the FCU and FDS. Based on these findings and the imaging results, and after discussion with the physician, ultrasound-guided hydrodissection (hydrorelease) was performed in the cubital tunnel and around the FDS branch of the median nerve. After the injection, pain decreased and muscle strength improved.

Continued intervention and final outcome

The same exercise therapy used during the initial phase (Videos 3, 4) was continued. Consequently, pain diminished and muscle strength recovered. Under non-weight-bearing conditions with valgus stress, ultrasonography demonstrated narrowing of the medial joint space during FDS contraction (Video 5). Two months after injury - after four physical-therapy sessions at two-week intervals - the patient successfully returned to competitive play. At four months, he continued to compete without reinjury.

**Video 5:** Functional evaluation of flexor digitorum superficialis (FDS) as a semi-dynamic stabilizer of the right elbow After intervention on the affected side, FDS contraction improved, and a reduction in widening during contraction was confirmed.

## Discussion

In this patient, the patient presented with a UCL injury accompanied by functional impairment of both the ulnar and median nerves. Through interventions aimed at improving the neuromuscular function of these nerves, the patient achieved a full return to competition within two months after injury. Typically, the return-to-play period following conservative treatment for UCL injuries is three to four months [9]. Although MRI and ultrasonography revealed structural damage and joint laxity of the UCL, the patient achieved an early return to play, which was noteworthy.

A key factor underlying this favorable outcome was the functional improvement of the FDS and FCU, both of which are essential dynamic stabilizers against valgus stress [5-7]. In this patient, the increased contractile force of the FDS and FCU contributed to narrowing the medial joint space opening, thereby enhancing dynamic elbow stability. The FDS innervated by the median nerve demonstrated strength recovery after ultrasound-guided identification of tender sites along the nerve, followed by repeated manual therapy and longitudinal/transverse gliding interventions, which reduced perineural tenderness and restored the muscle function. Similarly, ultrasonography-guided manual therapy and ulnar nerve mobilization improved pain and facilitated recovery from FCU.

Manual interventions targeting neural gliding effectively alleviate perineural tenderness and entrapment-related symptoms [10]. Therefore, the findings of this patient suggest that in instances where neurological symptoms accompany UCL injury, combining interventions for nerve function and innervated muscles may complement the intrinsic healing process of the ligament and enhance dynamic stability, thereby promoting an earlier and safer return to play.

This study had certain limitations. The degree of neural impairment was not confirmed using electromyography. In patients with more severe neurological deficits, the current treatment strategy may be insufficient. In addition, the report was limited to a single patient, and follow-up was restricted to four months, precluding the evaluation of long-term outcomes.

## Conclusions

This patient demonstrates that in a patient with UCL injury accompanied by ulnar and median nerve symptoms, ultrasound-guided manual therapy and neural mobilization effectively restored FDS and FCU function, enabling an earlier return to play with conservative treatment. These findings suggest that such interventions may represent a useful strategy for facilitating a safe and timely return to competition in throwing athletes with concomitant neural involvement.
